# Supplementary material for: Workshop, Cost-Effective and Streamlined Fabrications of Re-Usable World-To-Chip Connectors for Handling Sample of Limited Volume and for Assembling Chip Array
Source: Sensors (Basel). 2018 Dec 1;18(12):4223. doi: 10.3390/s18124223 (PMC6308506; doi:10.3390/s18124223)
Supplement: Supplementary file 1 [file sensors-18-04223-s001.pdf]

sensors

MDPI

Article

Workshop, Cost-Effective and Streamlined Fabrications of Re-Usable World-to-Chip Connectors for Handling Sample of Limited Volume and for Assembling Chip Array

Jiann-Hwa Lue, Yu-Sheng Su, and Tai-Chih Kuo

Supplementary Materials

Use of Templates in drilling holes on coverslips

To facilitate drilling holes on coverslips, we prepared a drilling template, made of a frame, thick glass, guide plate, and paper pad (Figure S1). Before drilling holes, a clean paper pad (4.8 × 7.8 mm) was placed above the glass pad, and the frame was lowered. A coverslip was then put in the rectangular space, and the guide plate was laid over. With a firm yet gentle press on the guide plate and the frame, the burr-tip of a running grinder was placed on the coverslip through the holes of guide plate. In a few seconds, a hole was made.

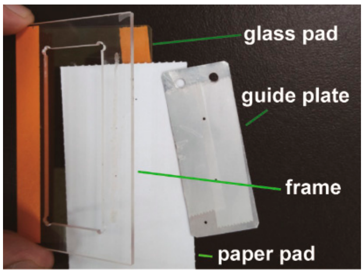

Figure S1. A template for drilling holes on coverslips. The frame was prepared by cutting out a rectangle (24 × 60 mm) from the center of a PMMA (4.8 × 7.8 × 2 mm) plate. The glass pad (4.8 × 7.8 × 3 mm) could be replaced with a PMMA plate. A guide plate was made by drilling holes on a rectangle aluminum strip (24 × 60 × 0.3 mm). A paper sticky tape (orange color) was used to hold the glass pad and the frame together. The aluminum strip can be replaced with a piece of transparency film.

The Key of Obtaining Clean Cuts of Double Adhesive and Transparency Films

Many units of holes and channels can be drawn in one double adhesive film (and transparency film) (for example, Figure S2a). However, to obtain a sheet of stamplets like our result (Figure S2b), a direct and non-stop cut of a film often ends with failures. It is because the debris from cut film might attach and accumulate around the blade. The debris could jam the blade, peel skin from the double adhesive film, and cause wrinkles of the film. To prevent these problems, the cuts are divided into groups on separated Adobe Illustrator layers. For example, the drawings shown in Figure S2a was separated into six layers. L1 and L2 contained horizontal lines, L3 vertical ones, and L4~L6 channels and holes (Figure S2c). At the end of cutting one layer, the cut can be held before going to the next layer. Moreover, each layer can have its own cutting parameters (cutting forces, repeats, and patterns). For both double adhesive film and transparency film, we set the media type as 'thick media', yet use a rad blade cap to cut double adhesive films, and a yellow one for transparency films. The cutting speed was set at 2 cm/s.

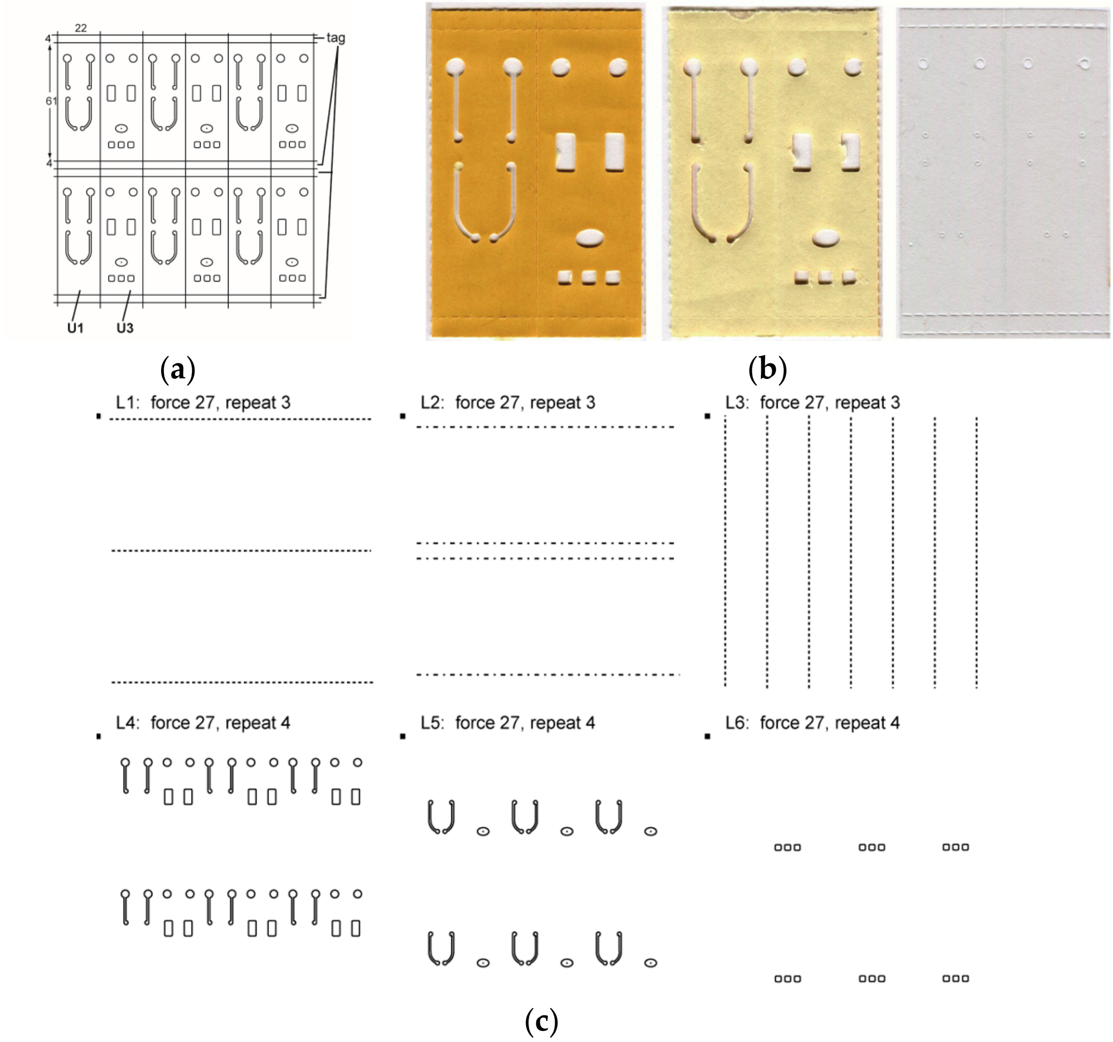

Figure S2. The designs and cuts of channels and holes of films used in the immobilization of GFP protein. (**a**) The designs of channels and holes of Unit 1 (U1) and Unit 3 (U3) of double adhesive films. Note that each unit has two tags for aligning layers of double adhesive films and transparency films (see main text Figure 2a). (**b**) The examples of cut double adhesive films (left and middle) and transparency film (right) in pairs. Double adhesive film has two skin, one with brown color (left), and the other light yellow (middle). (**c**) The lines, holes, and channels shown in the panel A are divided into six Adobe Illustrator layers, L1~L6. The square at the upper left corner at each layer serves as the reference mark.

The Design and Fabrication of Lab-Chip-For-Fish and Bridge

The design of PMMA plate, double adhesive films and transparency films of fish chip and bridge are illustrated in Figure S3.

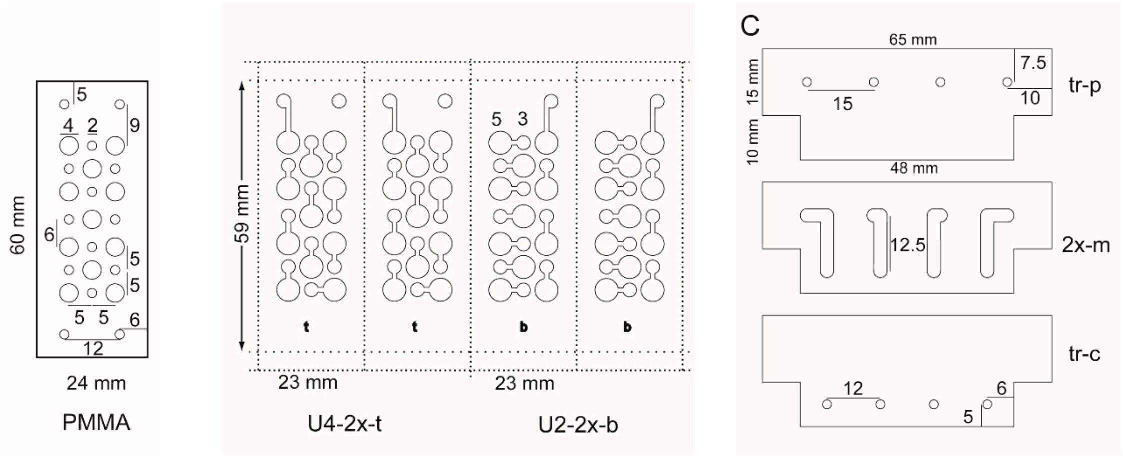

(**a**) (**b**) (**c**)

Figure S3. The specifications of PMMA plate, double adhesive films, and transparency films of fish chip and bridge. (**a**) PMMA plate (60 × 24 × 3 mm) with large (4 mm) and small (2 mm) holes. (**b**) The double adhesive films of fish chip. U4-2x-t and U2-2x-b are the Craft Robo-cut films which delineate the fluidic channels on the top and bottom of the fish chip, respectively. Notice that the length and width of the double adhesive films are slightly smaller than that of PMMA plate; the diameters of holes of double adhesive films are larger than those in the PMMA plate. (**c**) The transparency (tr-p and tr-c) and double adhesive films (2x-m) of the bridge between the fish chip and port. The numbers (in mm) indicate the distances between holes or from edge.

The Alignments and Pile-Up of Units of Double Adhesive Films and Transparency Film

An alignment template (Figure S4) is very useful for precisely piling up units of films over glass slide. The alignment template can be made easily. On one side of the film support, two parallel transparency strips were adhered (with double adhesive tape) in the center and with 22 mm apart (Figure S4a). Also, a dash line was printed in red to mark the top of alignments. The film support was used to bring a unit of film to the vicinity of the desired position in the assembled chip. The slide frame (Figure A4b) was used to fix a glass slide in the center of the holder (Figure S4c). In addition to the template, a Scotch tape and dental bent tweezer will be needed. In using a piece of Scotch tape, it is important to fold one end back to form a non-sticky handle. The dental bent tweezer will be used to remove the pilled units from the film support (and the slide frame). Gloves should be worn to prevent leaving finger prints on the chip. The assembly of a microfluidic chip is described below (Figure S5).

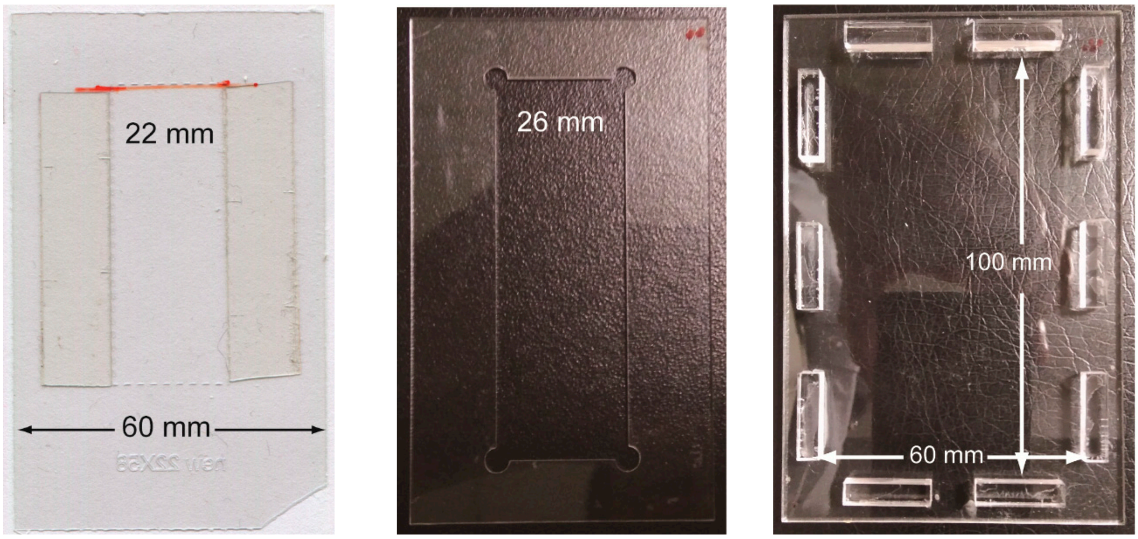

(**a**) (**b**) (**c**)

Figure S4. The parts of an alignment template. (**a**) Film support; (**b**) Slide frame; and (**c**) Holder.

The Stacking of Units of Double Adhesive and Transparency Films

In the first step (Figure S5a), the yellow skin of the tags of U1 film were peeled to expose the inner film where short pieces of Scotch tape were attached. The U1 film was placed on the support with the dashed lines of yellow skin of U1 aligned with the dashed lines of the support. With the yellow skin facing up, the U1 film was then attached to the support via Scotch tapes. The U1-attached support was placed into the holder (Figure S5b). The yellow skin of U1 was then removed with one adhesive surface exposed (Figure S5c). A glass slide was immobilized in the slide frame by three pieces of Scotch tapes (Figure S5d). The slide-containing frame was put in the holder with the film support already in place. A gentle press was applied to make U1 film adheres with the slide (Figure S5e). With the use of a dental bent tweezer, the film support was removed from the slide frame which now had a slide with U1 attached (Figure S5f). The U2 film was then attached onto the film support via tags and pieces of Scotch tape (Figure S5g). The U2-attached film support and the U1-slide containing frame were put into the holder in a back-to-back manner and with the film support on the top. The position of U2 film was repeatedly adjusted by unfastening and refastening the Scotch tape. Figure S5h shows a poor alignment of U2/U1 films. After a good back-to-back alignment was obtained, the U2-containing film support and the U1-slide containing frame were both taken out from the holder. The brown skin of U1 film (Figure S5i) was then removed. The U2-containing film support was put into the holder face-up, and then the U1-slide containing frame was placed down. A gentle yet firm press was applied to have the U2 film piled over the U1 on the slide (Figure S5j). Again, the film support was detached from the slide frame. The U3 film was loaded onto the film support and back-to-back alignments with U2-U1-slide were performed (Figure S5k). After obtaining a good alignment, the U3-film support was put into the holder, and the yellow skin of U3 film was removed (Figure S5l). Then, the U2-U1-slide in the slide frame was placed into the holder face-to-face with the U3 film to have the U3 film piled over the U2-U1-slide. The U3-U2-U1-slide was taken out from the holder with the brown skin of U3 film on the top (Figure S5m). The brown skin was then removed and replaced with a coverslip to complete the fabrication (Figure S5n, also main text, Figure 2b).

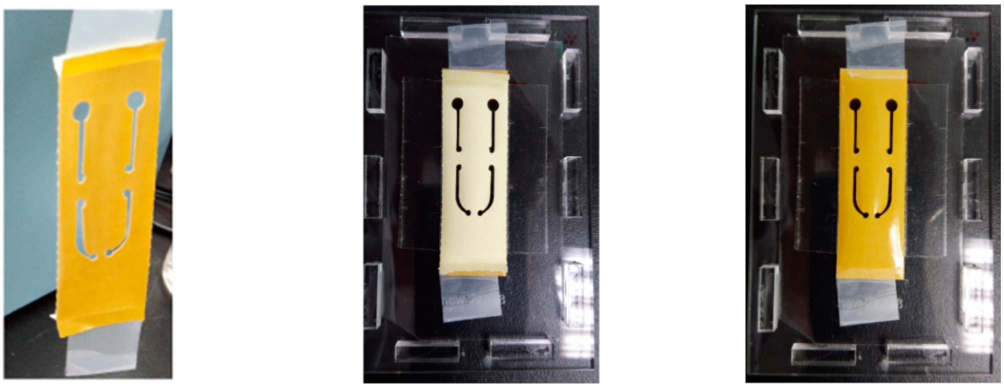

(**a**) (**b**) (**c**)

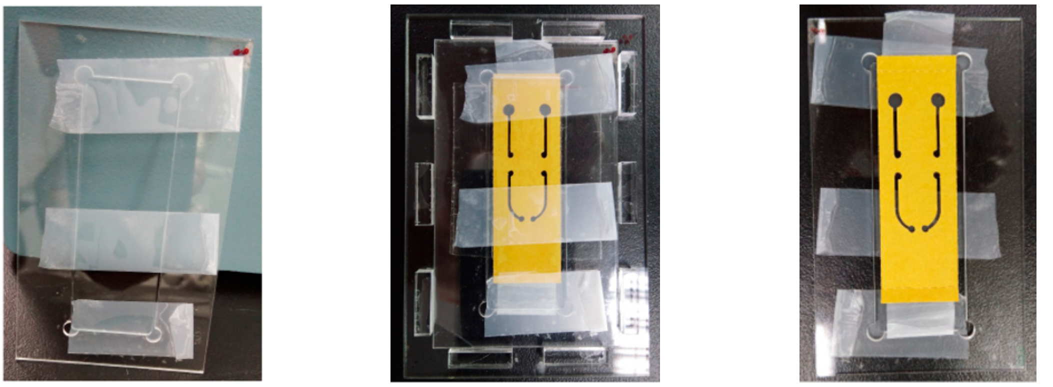

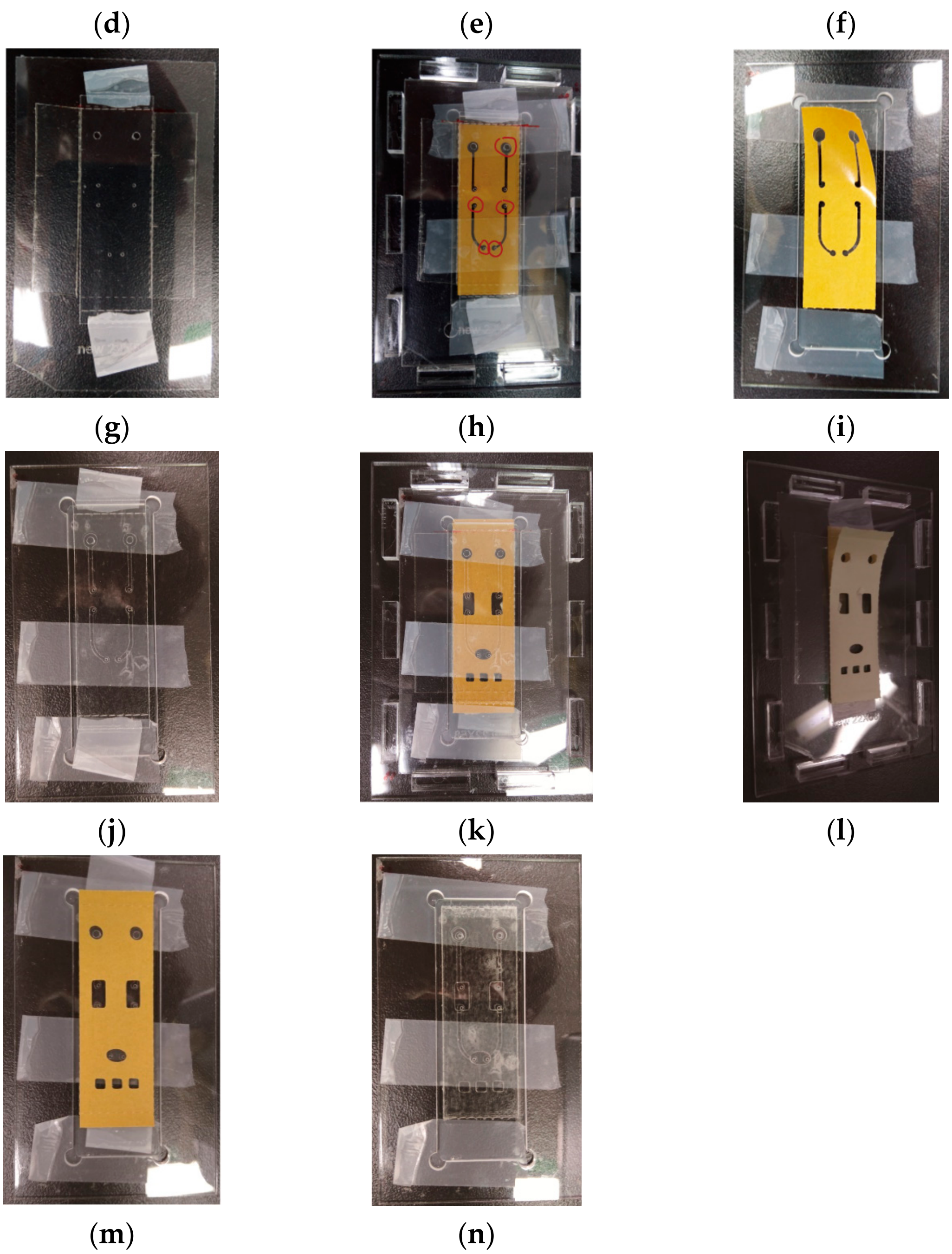

Figure S5. Pictures showing the steps of aligning and stacking units of double adhesive and transparency films to form a microfluidic chip.
